# Periocular Asymmetry Index in Caucasian Populations Using Three-dimensional Photogrammetry Assessment

**DOI:** 10.1007/s00266-024-04125-8

**Published:** 2024-05-28

**Authors:** Xiaojun Ju, Alexander C. Rokohl, Wanlin Fan, Michael Simon, Xueting Li, Xincen Hou, Nexhat Ukehajdaraj, Philomena A. Wawer Matos, Yongwei Guo, Ludwig M. Heindl

**Affiliations:** 1 Department of Ophthalmology, Faculty of Medicine, University Hospital Cologne, University of Cologne, Kerpener Straße 62, 50937 Cologne, Germany; 2 Center for Integrated Oncology (CIO) Aachen-Bonn-Cologne-Duesseldorf, Cologne, Germany; 3 Eye Center, The Second Affiliated Hospital, School of Medicine, Zhejiang University, Zhejiang Provincial Key Laboratory of Ophthalmology, Zhejiang Provincial Clinical Research Center for Eye Diseases, Zhejiang Provincial Engineering Institute on Eye Diseases, 88 Jiefang Road, 310009 Hangzhou, China

**Keywords:** Three-dimensional, Stereophotogrammetry, Periocular asymmetry, Age, Gender, Variation

## Abstract

**Objective:**

To quantitatively assess the periocular asymmetry and investigate its sex and age-related differences in a Caucasian population using three-dimensional (3D) stereophotogrammetry.

**Method:**

Standardized 3D photos of the periocular region of 301 Caucasians were taken using the VECTRA M3 3D Imaging System. Standardized landmarks were positioned, and data measurements in the periocular region were obtained from these images using VAM software and assessed using intraclass correlation coefficients (ICC) for reliability. Absolute differences and asymmetry index between the right and the left periocular region were calculated with a focus on gender and age-related differences.

**Result:**

Fissure height asymmetry was positively correlated with age (*p < 0.01*). Furthermore, reflex distance asymmetry was positively correlated with age in the elderly group (*p < 0.05*). Absolute differences of upper eyelid crease were 0.65 ± 0.79 mm for females and 0.90 ± 0.94 mm for males (*p < 0.05*). In the elderly group, the absolute differences of reflex distance were 0.664 ± 0.471 mm for males and 0.470 ± 0.408 mm for females (*p < 0.05*), while the absolute differences of fissure height were 0.619 ± 0.469 mm and 0.436 ± 0.372 mm, respectively (*p < 0.05*). All measurements had an inter-rater ICC and intra-rater ICC between 0.761 and 0.957.

**Conclusion:**

Periocular asymmetry is more prominent in older and male people. 3D photogrammetry is a reliable tool to detect periocular asymmetry and might provide an accurate and effective reference for periocular cosmetic, plastic, and reconstructive surgery in the clinical routine.

**Level of Evidence I:**

This journal requires that authors assign a level of evidence to each article. For a full description of these Evidence-Based Medicine ratings, please refer to the Table of Contents or the online Instructions to Authors. www.springer.com/00266.

## Introduction

A slight facial asymmetry is prevalent but unnoticeable in most humans. However, a certain degree of symmetrical facial features signifies good health and beauty. Previous studies showed that facial symmetry influences facial attractiveness, especially in males.[1] It has also been demonstrated that individuals with minor asymmetries are more consistent with the definition of beauty.[2] Although facial symmetry reflects molecular genetics and development, it can also be affected by environmental factors, nutrition, disease, and behavior.[3] Some common ophthalmic diseases, such as ptosis, can also affect periocular symmetry.[4] Periocular asymmetry, triggered by external ocular disease, not only affects the aesthetic appearance but also has a detrimental effect on visual function.[5, 6] Ptosis causes asymmetrical changes in facial expressions, such as brow lifting to widen the eyelid fissure. This unilateral or bilateral asymmetrical brow lift has functional and aesthetic consequences, such as deepening forehead lines, changing facial expression, and upsetting facial symmetry.[7, 8] Therefore, postoperative periocular symmetry is a crucial aesthetic and functional indicator.

Only a few studies have considered periocular asymmetry, whereas most previously published studies have concentrated on overall facial asymmetry. Due to the dynamic expressions in the bottom half of the face, asymmetries in the upper part (including the eyes, brows, and forehead) are very noticeable when the face is resting.[9] Previous studies have verified age and gender differences in orbital tissue symmetry.[10, 11] Therefore, there is an urgent need to study the asymmetric structure of the periocular external soft tissue to meet clinical needs. Direct measurement, 2-dimensional (2D) photogrammetry, and 3-dimensional (3D) stereophotogrammetry are the primary methods for obtaining anthropometric measures.[12, 13] Compared to direct measurement and 2D analysis, 3D measurement has the advantages of accuracy and reliability.[14–17] Although facial asymmetries in various ethnic groups have been recorded in previous studies, there is a dearth of 3D stereographic data describing periocular asymmetries, particularly in Caucasians.

This study aims to utilize 3D stereophotogrammetry imaging to measure the periocular region and provide a highly accurate and reliable database of periocular asymmetry in the Caucasian population through standardized landmark settings and quantitative analysis of 3D images. Meanwhile, this study presents for the first time some preliminary considerations about the periocular asymmetry index in the Caucasian population. It also provides data for reference in developing periocular cosmetic surgery, deformity diagnosis, and correction.

## Methods

### Study Volunteers

A total of 301 German Caucasian volunteers were consecutively recruited from May 2020 to December 2020 at the University Hospital of Cologne, Germany. The exclusion criteria were: (1) any orbital or ophthalmic comorbidities that could affect eyelid position, especially blepharochalasis, severe dermatochalasis, eyebrow ptosis, eyelid retraction, thyroid orbitopathy, entropion and ectropion, Horner’s syndrome, ocular trauma, or scarring in the periocular region. (2) Volunteers who could not cooperate with the examination due to age or physical reasons. (3) Volunteers wearing make-up. All the participants provided written informed consent. This study followed the principles of the Declaration of Helsinki and was approved by the Ethics Committee of the University of Cologne (approval number: 17–199). All patients provided informed consent for the publication of their data and images.

### 3D Photo Capture

The Vectra M3 system (America, Company Canfield Scientific), a three-pod passive stereo photogrammetry system with three cameras in fixed positions that captures all images simultaneously within 3.5 milliseconds, was used for 3D photogrammetry image collection in this study and measurement. An experienced examiner (YG) obtained all 3D images in a standardized manner using a VECTRA M3 3D stereo camera. The volunteers sat in front of the camera and maintained their heads in a natural position, with their eyes looking straight ahead. The camera simultaneously captured photos from the left, front, and right directions. The VECTRA Analysis Module software automatically synthesized the 3D photos. If the synthesis failed, shooting was repeated until successful. Demographic data, including sex, race, and age, were collected during imaging.

### Landmark Location and Data Measurement

**Fig. 1:**
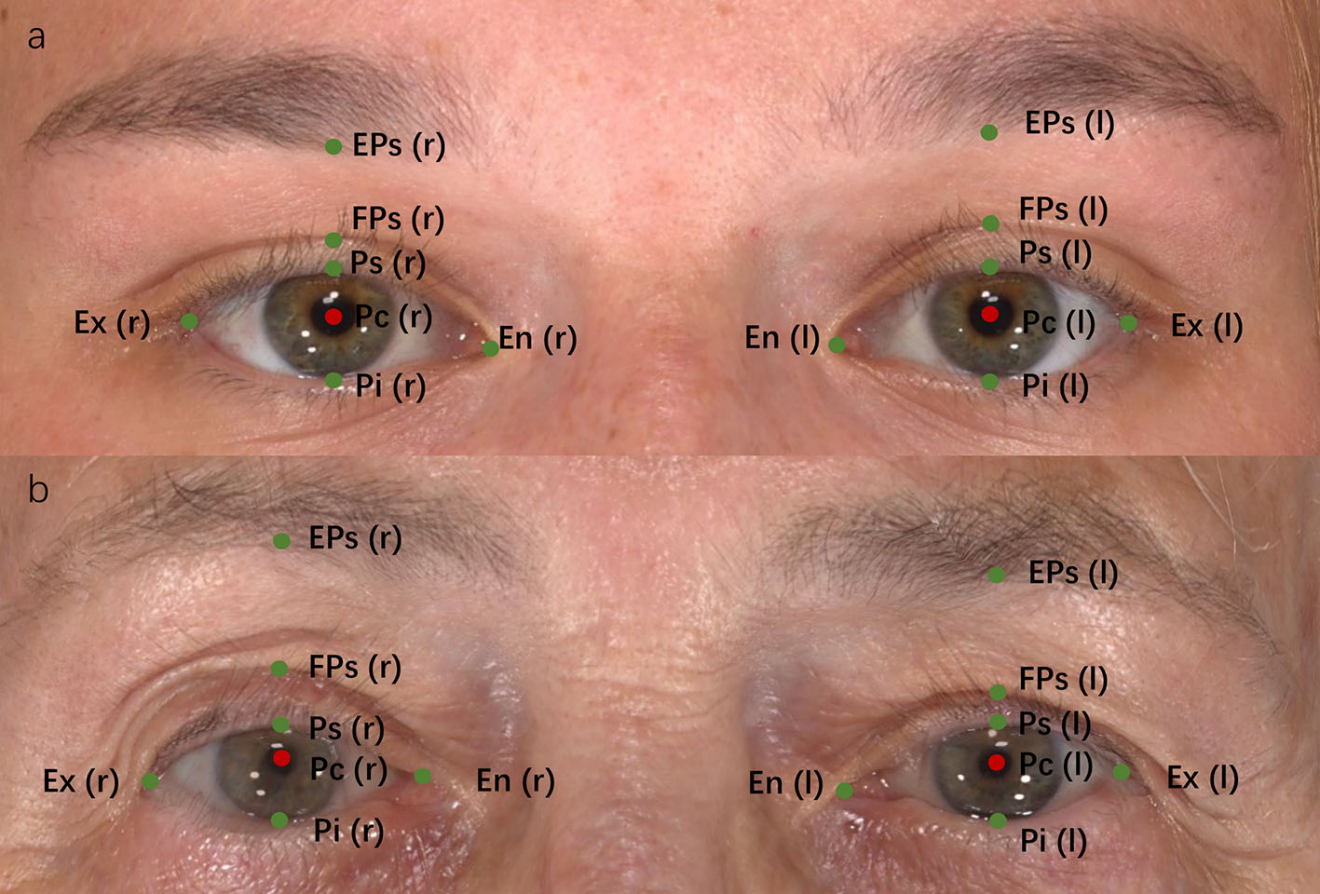
Periocular landmarks in female and male volunteers of different ages and gender: **a** A 24 years old female, **b** A 80-year-old female

**Fig. 2:**
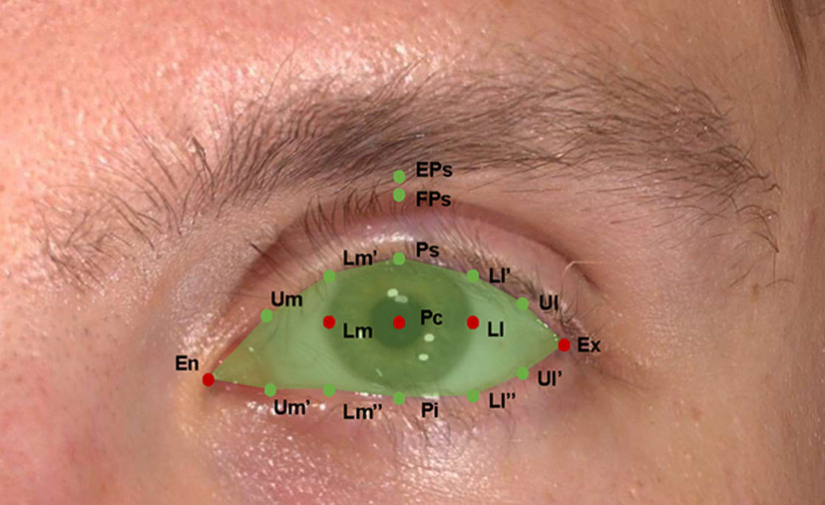
Periocular landmarks for measuring the area of ocular surface exposure

Bilateral periocular landmarks were marked on each 3D photograph, as shown in Table 1**.** Male and female volunteers were divided into young (≤ 35 years old), middle-aged (35 to 60 years old), and elderly ( > 60 years old) groups according to age, and ocular surface landmarks were marked on each 3D photo (Figure 1). The measurement indicators in the coronal view were: (1) reflex distance: the distance from the center of the pupil to the vertical corresponding upper eyelid margin; (2) fissure height: the distance between the upper and lower eyelid margins corresponding vertically to the center of the pupil; (3) upper eyelid crease: vertically corresponds to the center of the pupil, the distance from the upper eyelid point on the same side to the eyelid crease point; (4) pupil-to-brow: the vertical distance from the center of the pupil to the lower edge of the eyebrow on the same side and (5) ocular surface exposed area: the uncovered area of the eyeball surrounded by the upper and lower eyelids (Figure 2).

**Table 1:** Definitions of anthropometric landmarks and parameters in the periocular region

Landmark	Liner distance
En	The upper and lower eye edges meet at the inner corner of the eyes
Ex	The upper and lower eye edges meet at the outer corners of the eyes
Ps	Point vertical to Pc at the upper palpebral margin on the eyelid edge
Pc	The center point of the pupil
Pi	Point vertical to Pc at the lower palpebral margin on the eyelid edge
Lm	Medial corneoscleral limbus point horizontal to pupillary center
Ll	Lateral corneoscleral limbus point horizontal to pupillary center
FPs	Point vertical to Pc at the upper eyelid crease
EPs	Point vertical to Pc at the lower margin of the eyebrow
Lm’	Point vertical to Lm at the upper palpebral margin on the eyelid edge
Lm”	Point vertical to Lm at the lower palpebral margin on the eyelid edge
Ll’	Point vertical to Ll at the upper palpebral margin on the eyelid edge
Ll”	Point vertical to Ll at the lower palpebral margin on the eyelid edge
Um	Middle point between En and Lm’ at the upper palpebral margin on the eyelid edge
Um’	Middle point between En and Lm” at the lower palpebral margin on the eyelid edge
Ul	The middle between Ex and Ll’ at the upper palpebral margin on the eyelid edge
Ul’	The middle between Ex and Ll” at the lower palpebral margin on the eyelid edge
Reflex distance (Ps–Pc)	The distance between Ps and Pc
Fissure height (Ps–Pi)	The distance between Ps and Pi
Upper eyelid crease (Ps–FPs)	The distance between Ps and FPs
Pupil-to-brow (Pc–EPs)	The distance between Pc and EPs
Ocular surface exposed area (En-Um-Lm’–Ps- Ll’- Ul- Ex- Ul’- Ll”- Pi- Lm”- Um’)	The area enclosed by the upper and lower eyelids

The asymmetry between the right and left periocular regions was analyzed using absolute difference values. According to a previous study [18, 19], the asymmetry index was calculated using the absolute value of the bilateral difference as follows: absolute two-sided difference/smaller values on both sides×100%.

### Statistical Analysis

To evaluate inter-rater reliability, two raters (rater 1 [XJ] and rater 2 [NU]) independently performed image measurements and analyses. After an interval of at least 24 h, rater 1 (XJ) performed a second measurement and analysis of the same images to assess intra-rater reliability. Intraclass correlation coefficients (ICCs) were used to assess reliability. The degree of agreement was considered poor if ICC < 0.40, satisfactory if 0.40 ≤ ICC < 0.75, and excellent if 0.75 ≥ICC [20].

IBM SPSS Statistics 27 (IBM Corp., Armonk, NY, IBM Corp.) was used for the statistical analysis. All figures were generated using GraphPad Prism 8.0.1 (GraphPad Software, San Diego, CA, USA). All data maintained a normal distribution, as verified by the Kolmogorov–Smirnov test. A paired t-test was conducted to analyze whether the asymmetry was statistically different, and Pearson's rank correlation coefficient was used for correlation analysis. The absolute bilateral differences and asymmetry index for each measurement item were analyzed further. The Mann–Whitney U-test was performed to evaluate statistical sex differences. Statistical significance was considered at *p* < 0.05.

## Results

A total of 301 volunteers were recruited for this study. All volunteers were German Caucasians, including 128 men (51.2 ± 19.2 years old) and 173 women (54.1 ± 19.5 years old) (*p* = 0.215). They were divided into three age groups: The young group included 38 men (28.4 ± 3.9 years old) and 47 women (28.5 ± 3.7 years) (*p* = 0.880), the middle-aged group included 45 men (48.3 ± 7.6 years) and 53 women (49.5 ± 6.6 years) (*p* = 0.547), while the elderly group included 45 men (72.3 ± 7.7 years) and 73 women (72.9 ± 7.2 years) (*p* = 0.589). No statistically significant age differences were identified between men and women (*p* > 0.05).

### Coronal Plane Measurement of Asymmetry

The measurement results (mean and standard deviation) of the coronal plane and ocular asymmetry index are shown in Table 2. By establishing periocular coordinate axes, we performed 3D measurements of the ocular surface exposed area, reflex distance, fissure height, upper eyelid crease, and pupil-to-brow. No significant differences were found between the left and right reflex distance, fissure height, upper eyelid crease, pupil-to-brow, or ocular surface exposed area (*p* ≥ 0.05, respectively). Further analysis of the asymmetry index at different measurement points revealed that upper eyelid crease had the highest asymmetry index of 18.55%, 22.54% in males, and 16.52% in females. Fissure height had the smallest asymmetry index of 5.59%, 6.48% in males and 4.93% in females. At the individual data level, it can be seen that there is left-side dominance in reflex distance, upper eyelid crease, and pupil-to-brow and right-side dominance in fissure height and ocular surface exposed area.

**Table 2:** Measurements of the periocular parameters in the coronal plane

Parameters	Total volunteers (n = 301)				Male volunteers (n = 128)				Female volunteers (n = 173)			
	Right	Left	Asymmetry index	*p*-value	Right	Left	Asymmetry index	*p*-value	Right	Left	Asymmetry index	*p*-value
Reflex distance (mm)	4.61±0.74	4.66±0.78	13.12%	0.203	4.6±0.82	4.61±0.78	14.83%	0.865	4.62±0.68	4.71±0.77	11.85%	0.108
Fissure height(mm)	9.28±1.24	9.28±1.23	5.59%	0.957	9.51±1.16	9.44±1.1	6.48%	0.289	9.11±1.28	9.16±1.30	4.93%	0.207
Upper eyelid crease(mm)	4.43±1.53	4.48±1.58	18.55%	0.424	4.29±1.65	4.35±1.55	22.54%	0.609	4.52±1.44	4.57±1.60	16.52%	0.534
Pupil-to-brow(mm)	16.71±2.91	16.84±2.86	7.13%	0.099	15.69±2.45	15.85±2.24	7.66%	0.204	17.46±3.01	17.58±3.05	6.73%	0.279
Ocular surface exposed area (mm^2^)	1.85±0.40	1.84±0.39	8.65%	0.756	1.93±0.35	1.92±0.32	8.32%	0.812	1.79±0.42	1.78±0.41	8.95%	0.823

### Asymmetry Between Different Ages and Genders

**Fig. 3:**
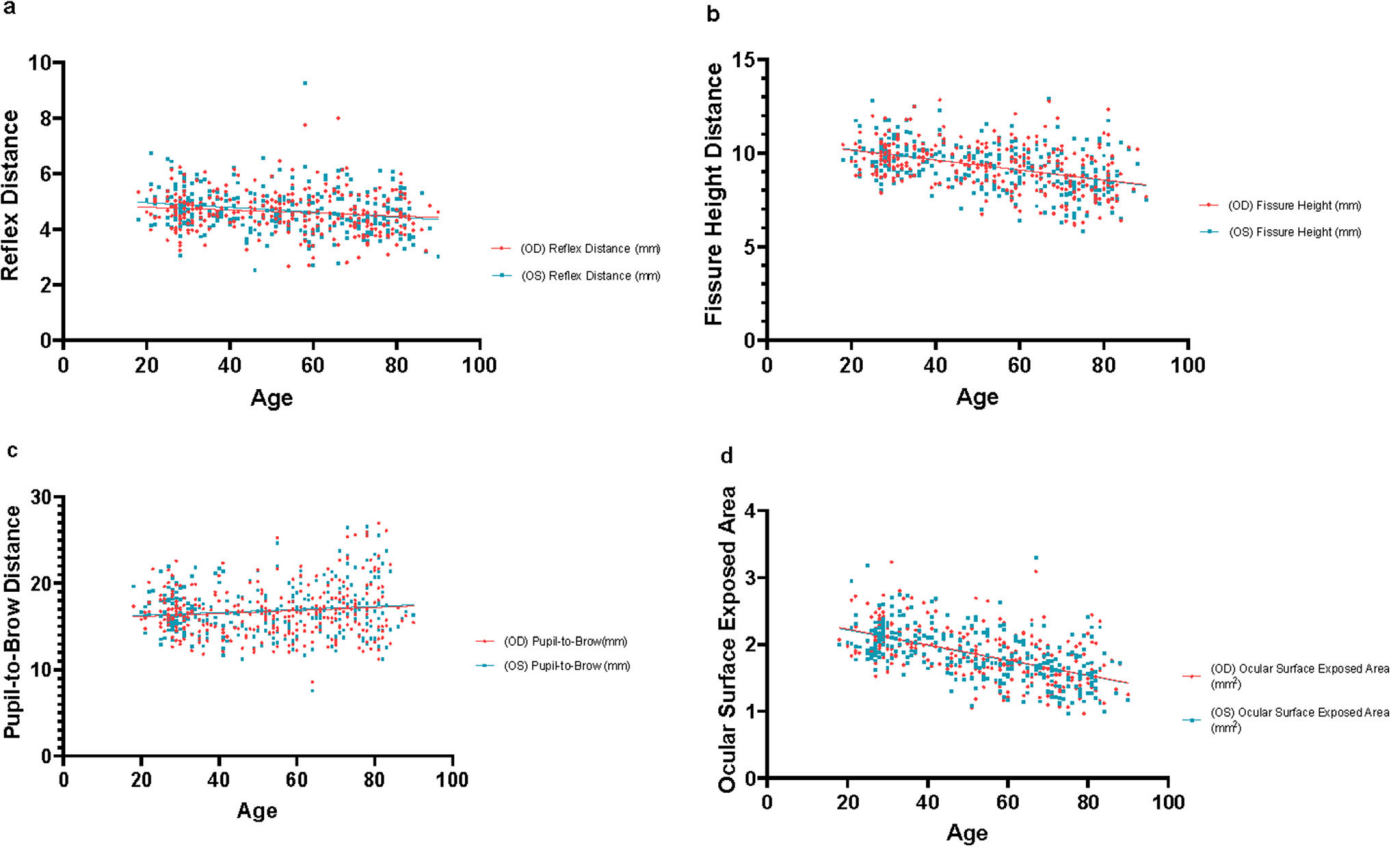
**a** Pearson correlation between reflex distance linear distance and age *(OD:P = 0.0210; OS:P = 0.0002)*, **b** Pearson correlation between fissure height linear distance and age *(OD:P < 0.0001; OS:P < 0.0001)*, **c** Pearson correlation between pupil-to-brow linear distance and age *(OD:P = 0.0443; OS:P = 0.0410)*, **d** Pearson correlation between the area of the ocular surface exposure and age *(OD:P < 0.0001; OS:P < 0.0001)*

**Fig. 4:**
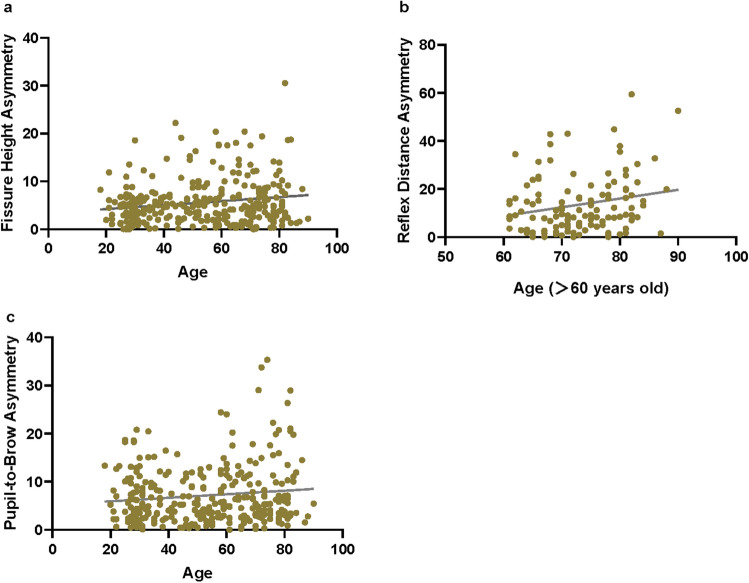
**a** Pearson correlation between fissure height asymmetry and age *(p = 0.0029)*, **b** In the elderly population, Pearson correlation between reflex distance asymmetry and age *(p = 0.0204)*, **c** Pearson correlation between pupil-to-brow asymmetry and age *(p = 0.0412)*

Based on 3D measurements of coronal plane landmarks, for the reflex distance linear distance, fissure height linear distance, and ocular surface exposed area, age was negatively correlated with the linear distance of the bilateral reflex distance, fissure height, and ocular surface exposed area (*p <* 0.05, respectively, Figure 3*).* Conversely, pupil-to-brow linear distance, and age were positively correlated** (***p* < 0.05, respectively*,* Figure 3).The subgroup analyses based on reflex distance, pupil-to-brow, and fissure height demonstrated a significant increase in bilateral asymmetry with age. fissure height and pupil-to-brow asymmetries were positively correlated with age (*p <* 0.05). In the older adults’ group, reflex distance asymmetry was positively correlated with age (*p* < 0.05). **(**Figure 4**).** Simultaneously, based on the three groups divided by age above, we further analyzed the left and right bilateral indices and asymmetry rates for the three age groups. (Table 3) In the young group, we can observe that in the measurements of reflex distance and fissure height, it is significant that the values of the left eye are higher than those of the right eye (*p* < 0.01, *p* < 0.05, respectively). In the middle-aged and elderly groups, the reflex distance, fissure height, and upper eyelid crease showed larger values on the right side, but they were not statistically significant. Regarding the asymmetry index, the corresponding reflex distance, fissure height, upper eyelid crease, pupil-to-brow, and ocular surface exposed area asymmetry index showed that the older group was the largest, followed by the middle-aged group and the young group. (Table 3)

**Table 3:** Measurements of the periocular parameters in different age groups

Parameters	Young (n = 85)				Middle aged (n = 98)				Elderly (n = 118)			
	Right	Left	Asymmetry index	*p*-value	Right	Left	Asymmetry index	*p*-value	Right	Left	Asymmetry index	*p*-value
Reflex distance (mm)	4.67±0.62	**4.90±0.68**	11.26%	0.000**	4.68±0.77	4.64±0.87	12.17%	0.705	4.52±0.78	4.52±0.72	13.57%	0.937
Fissure height (mm)	9.86±0.93	**9.99±0.98**	4.11%	0.014*	9.41±1.10	9.30±1.10	6.16%	0.108	8.74±1.33	8.74±1.22	6.19%	0.927
Upper eyelid crease (mm)	4.27±0.95	4.41±1.13	18.19%	0.269	4.33±1.83	4.49±1.66	18.25%	0.211	4.61±1.60	4.52±1.78	18.56%	0.339
Pupil-to-brow (mm)	16.64±2.19	16.84±2.09	6.16%	0.164	15.96±2.49	16.07±2.51	6.27%	0.373	17.37±3.50	17.49±3.42	8.04%	0.454
Ocular surface exposed area (mm2)	2.11±0.31	2.12±0.30	7.21%	0.833	1.90±0.36	1.87±0.35	9.15%	0.121	1.62±0.35	1.62±0.35	9.25%	0.996

Bold values are statistically significant (*p* < 0.05)

**p < 0.05, *p < 0.01

**Fig. 5:**
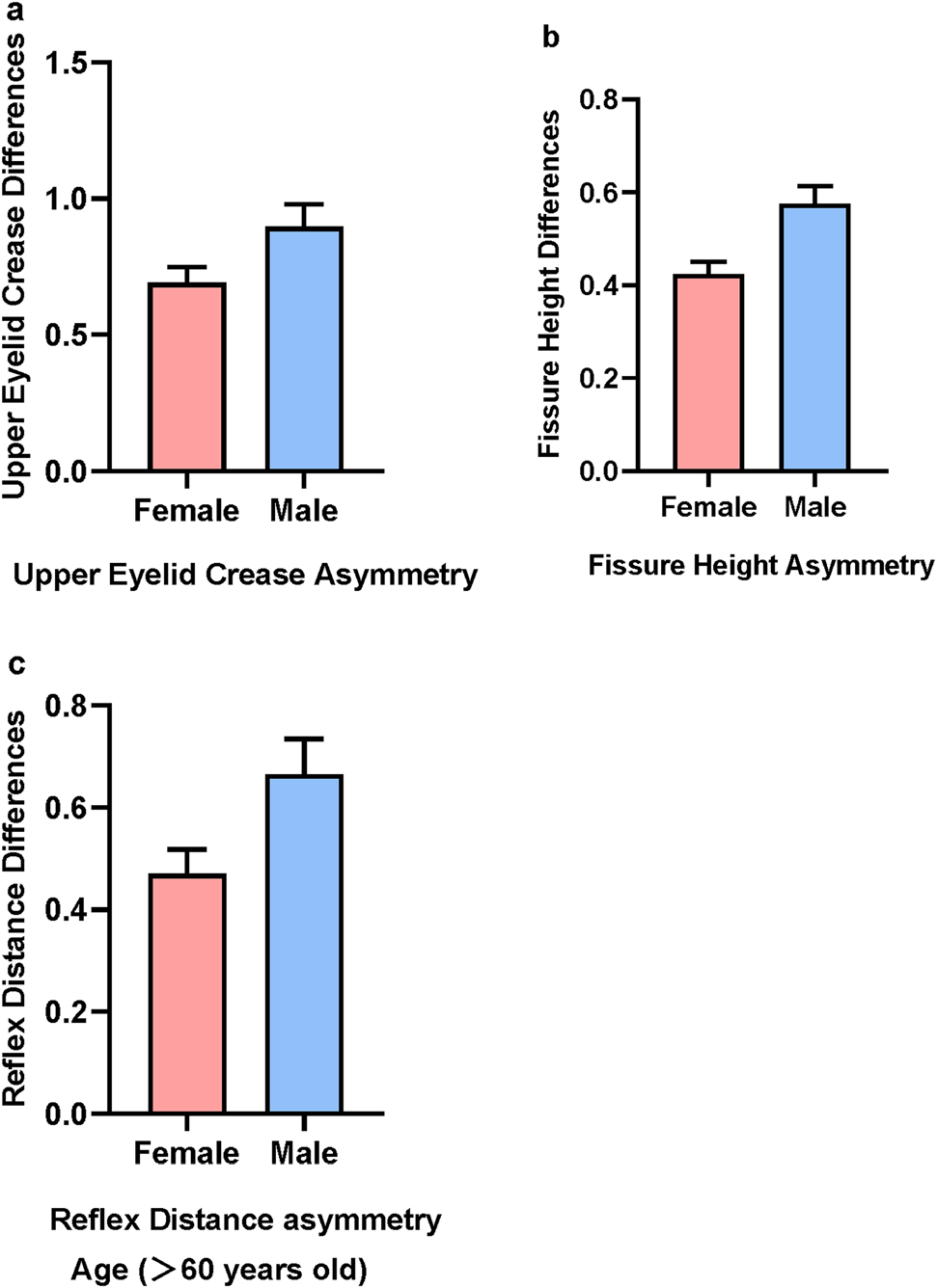
**a** Mann-Whitney U-test between upper eyelid crease asymmetry and gender *(p = 0.0424)*. **b** Mann-Whitney U-test between fissure height asymmetry and gender *(p = 0.0011)*. **c** In the elderly population, Mann-Whitney U-test between reflex distance asymmetry and gender *(p = 0.0172)*.

As for the symmetrical differences in gender, the absolute difference in upper eyelid crease was 0.65 ± 0.79 mm in women and 0.90 ± 0.94 mm in men (p < 0.05). In the older adults’ group, the absolute difference in reflex distance was 0.664 ± 0.471 mm in men and 0.470 ± 0.408 mm in women (p < 0.05); the absolute difference in fissure height was 0.619 ± 0.469 mm in men and 0.436 ± 0.372 mm in women (*p* < 0.05). (Figure 5). We also observed that in terms of asymmetry index, men’s reflex distance, fissure height, upper eyelid crease, and pupil-to-brow were 14.83%, 6.48%, 22.54%, and 7.66%, which were all higher than the corresponding indexes for women of 11.85%, 4.93%, 16.52%, and 6.73 %, and women’s asymmetry index in ocular surface exposed area is 8.95%, which is higher than men’s 8.32%.

### Observational Error

**Fig. 6:**
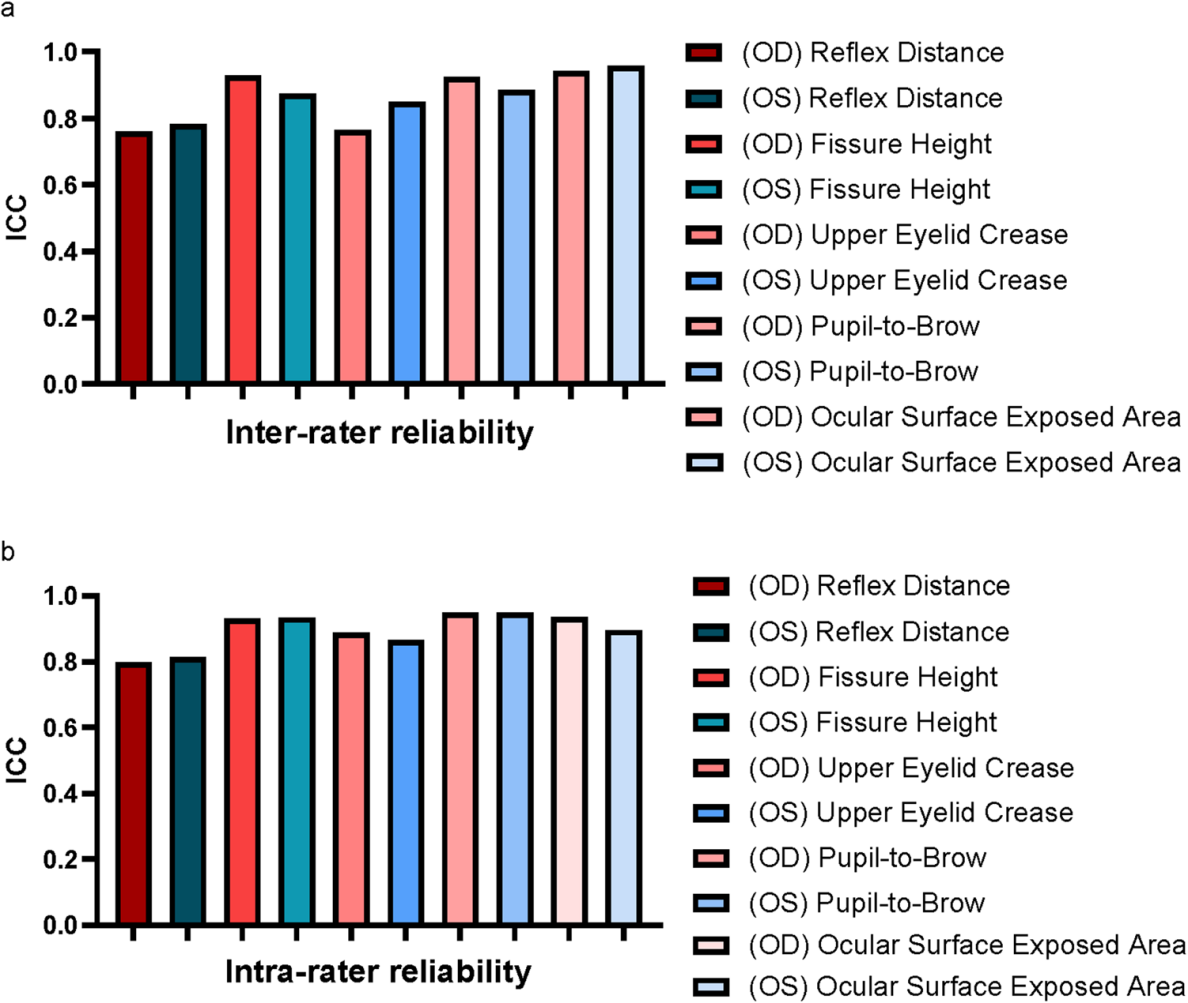
**a** The intraclass correlation coefficient for inter-rater, **b** The intraclass correlation coefficient for intra-rater

During the evaluation of inter-rater and inter-rater reliability, all ICC values were > 0.75 **(**Figure 6**)**. This result indicates that the anthropometric measurements in this study were reliable.

## Discussion

This study confirmed that the periocular area of Caucasians tends to be symmetrical, but subtle asymmetry is still common, and the degree of this asymmetry was quantitatively described by 3D measurement. The left–right linear distance difference between eyebrow position and eyelid height was within the range of the previously described difference in perceived asymmetry (eyebrow: 3.5 mm, eyelid: 1 mm).[8] This indicates that the periocular structures of ordinary individuals tend to be symmetrical within the range of perception. Therefore, to further analyze the degree of asymmetry in the periocular area, we propose to use the asymmetry index to quantify this bilateral periocular difference.

According to the results of this study, morphological changes in the periocular region are significantly influenced by age. As age increases, the linear distance of reflex distance and fissure height is negatively age-correlated, while the left–right asymmetry of the reflex distance and fissure height becomes more pronounced. We found that the asymmetry of fissure height increased with age, while that of reflex distance increased significantly in older adults. Previous research on facial symmetry has found that facial asymmetry in the upper third increases with age.[21] The results of our analysis are similar and refined in the asymmetry index. Comparing the asymmetry index of various periocular parts in different age groups, we found that the elderly (over 60 years old) are the most asymmetric compared with the middle-aged and young groups. The periocular asymmetry index is the largest among the population. Thus, among the changes in asymmetry around the eyes caused by age, the asymmetry index intuitively reflects this trend.

Aging causes increased skin folds but does not affect the position of the pupil center.[22] Thus, skin laxity and increased folds reduce the distance between the pupil and the corresponding upper eyelid. Fezza *et al*. found a linear increase in the lower eyelid length with age. That is, the inferior orbital rim descends as a whole with age.[23] This might be related to remodeling of the facial skeleton and expansion of the bony sulcus with age. As the infraorbital rim drops, the overlying soft tissues attached to the skeleton, such as the septum and the orbicularis muscle, are pulled down, resulting in a change in the position of the lower eyelid.[24] Such changes, however, focus on a slight increase in the horizontal length of the external canthus caused by the thinning of the orbicularis oculi and fat prolapse.[25, 26] We noted a significant decrease in fissure height with age, and aging can result in a downward shift of the upper and lower eyelids, as well as a reduction in the lid fissure height. This raises the question of whether this indicates that the laxity of the upper eyelids changes more with age than that of the lower eyelids, perhaps to be answered by further cohort observations of periocular morphology.

Gender is significantly related to periocular asymmetry. Men had a higher upper eyelid crease asymmetry than women. A Japanese study reported that prevalence of upper eyelid asymmetry increases in Japanese adults. The patterns in the asymmetrical upper eyelid folds included lateral (left side > right side only) and sex (female > male) differences.[27] In contrast to most Asians with no or reduced upper eyelid folds[28], all patients in our study were Caucasians; therefore, anatomical differences contributed to the differences in upper eyelid crease asymmetry. Older adults have the most altered facial morphology of all changes in facial structure, involving changes in bone, fat, and skin morphologies. Lambros *et al.* found that upper lid ptosis and upper lid arc shift are seen clearly as one to two-millimeter shifts from youth to old age.[29] This aging tendency exacerbates periocular asymmetry. Among the 118 older adults in our study cohort, men were significantly more likely to have reflex distance and fissure height asymmetries than women. In previous observational studies on periorbital aging, men aged > 40 years were more likely to have sagging eyelid skin than women.[30] This reason is potentially related to women maintaining their appearance through makeup maintenance and long-term control of facial expressions.

There is a clear correlation between unconscious asymmetrical eyebrow lifts and ocular dominance.[31] A small amount of asymmetry in healthy people can still be aesthetically pleasing. Still, it has been noted that asymmetry can be more pronounced and unpleasant if the periocular areas are not youthful.[32] We found that the linear distance of the pupil-to-brow increases with age, while eyebrow asymmetry increases with age, and we speculate that the dominant eye and associated muscles may contribute to this aging change. In addition, a survey of patients with ptosis undergoing surgical treatment showed that both patients and surgeons expected an improvement in their asymmetric brow position after surgery.[8] Therefore, the symmetry between the eyebrows can be used as an important criterion for symmetry adjustment in periocular plastic and cosmetic surgeries.

This study found that the inter- and intra-rater ICCs were > 0.75, indicating that the measured data were reliable. Therefore, 3D photogrammetry makes it feasible and reliable to measure the periocular region and evaluate periocular symmetry. 3D photogrammetry has increasingly become a routine measurement method for evaluating patients undergoing orthodontic, oral, and plastic surgery. This technique has been rapidly adopted for its ability to perform non-contact, accurate, spatially intensive, automated, and rapid coordinate measurements of facial soft tissues. Our previous studies have shown that 3D photogrammetry can be used to reliably perform periocular linear, areal, and volumetric measurements [14, 15, 33–35] and evaluate eyelid functional changes. [36–38] Therefore, we continued the previous method in this study by establishing landmarks, quantifying the basic periocular linear distance and describing the symmetry.

A research topic in recent years has been how to quantify the periocular region better and maintain periocular aesthetics. 2D photographs have been used clinically to perform periocular measurements for a long time.[39, 40] We used reliable 3D photogrammetric data to analyze periocular symmetry in Caucasian populations quantitatively. It provides baseline anthropometric and periocular asymmetry data on a large sample that can be used to plan periocular surgery and evaluate postoperative outcomes. Simultaneously, 3D image recording can make the patient intuitively aware of the existing periocular asymmetry before surgery. Surgeons can track a patient’s disease progression within this range through surgical interventions based on preoperative assessments. Based on 3D measurements, we calculated and analyzed the periocular asymmetry index in the Caucasian population for the first time, which can bring a new reference for clinical assessment of the severity of periocular asymmetry.

However, this study has some limitations. First, the recruitment of an adequate sample size must be improved, mainly because groups of children and adolescents did not participate. A more comprehensive sample size would further support the association between age and asymmetry. Additionally, our study only included landmark measurements in the coronal plane of the periocular area, whereas surface depth measurements, i.e., landmark measurements in the sagittal plane, are also necessary. Therefore, relatively more planar 3D photographic periocular area studies are required in the future. Furthermore, our study only analyzed the asymmetry of the periocular region in a Caucasian population and the relationship between the bilateral periocular asymmetry and age-gender, lacking an analysis of the aesthetic assessment of this asymmetry. In the future, we aim to assess further the details of periocular asymmetry on its aesthetic evaluation.

## Conclusions

This study provides a reproducible and reliable method for periocular asymmetry analysis based on 3D photogrammetry. The 3D measurements also offer quantitative local information on the symmetry of the periocular region. The periocular asymmetry index can provide new ideas for diagnosing diseases, planning treatment, and evaluating patients' periocular morphology. Furthermore, this study confirmed that the left and right periocular regions tend to be roughly symmetrical in Caucasian populations. However, the difference between the left and right periocular asymmetry still persisted and was affected by age and gender.
